# Mental health outcomes at the end of the British involvement in the Iraq and Afghanistan conflicts: a cohort study

**DOI:** 10.1192/bjp.2018.175

**Published:** 2018-12

**Authors:** Sharon A. M. Stevelink, Margaret Jones, Lisa Hull, David Pernet, Shirlee MacCrimmon, Laura Goodwin, Deirdre MacManus, Dominic Murphy, Norman Jones, Neil Greenberg, Roberto J. Rona, Nicola T. Fear, Simon Wessely

**Affiliations:** 1 Lecturer in Epidemiology, King's Centre for Military Health Research, UK; 2 Senior Research Associate, King's College London, UK; 3 Project Manager, King's College London, UK; 4 Database Administrator, King's College London, UK; 5 Database Administrator, King's College London, UK; 6 Lecturer in Epidemiology, University of Liverpool, UK; 7 Senior Clinical Lecturer, King's College London, UK; 8 Senior Clinical Lecturer, King's College London, UK; 9 Military Senior Lecturer, King's College London, UK; 10 Professor of Defence Mental Health, King's College London, UK; 11 Professor of Public Health Medicine, King's College London, UK; 12 Professor of Epidemiology, King's College London, UK; 13 Professor of Psychological Medicine, King's College London, UK

**Keywords:** Alcohol misuse, combat, common mental disorders, deployment, post-traumatic stress disorder

## Abstract

**Background:**

Little is known about the prevalence of mental health outcomes in UK personnel at the end of the British involvement in the Iraq and Afghanistan conflicts.

**Aims:**

We examined the prevalence of mental disorders and alcohol misuse, whether this differed between serving and ex-serving regular personnel and by deployment status.

**Method:**

This is the third phase of a military cohort study (2014–2016; *n* = 8093). The sample was based on participants from previous phases (2004–2006 and 2007–2009) and a new randomly selected sample of those who had joined the UK armed forces since 2009.

**Results:**

The prevalence was 6.2% for probable post-traumatic stress disorder, 21.9% for common mental disorders and 10.0% for alcohol misuse. Deployment to Iraq or Afghanistan and a combat role during deployment were associated with significantly worse mental health outcomes and alcohol misuse in ex-serving regular personnel but not in currently serving regular personnel.

**Conclusions:**

The findings highlight an increasing prevalence of post-traumatic stress disorder and a lowering prevalence of alcohol misuse compared with our previous findings and stresses the importance of continued surveillance during service and beyond.

**Declaration of interest:**

All authors are based at King's College London which, for the purpose of this study and other military-related studies, receives funding from the UK Ministry of Defence (MoD). S.A.M.S., M.J., L.H., D.P., S.M. and R.J.R. salaries were totally or partially paid by the UK MoD. The UK MoD provides support to the Academic Department of Military Mental Health, and the salaries of N.J., N.G. and N.T.F. are covered totally or partly by this contribution. D.Mu. is employed by Combat Stress, a national UK charity that provides clinical mental health services to veterans. D.MacM. is the lead consultant for an NHS Veteran Mental Health Service. N.G. is the Royal College of Psychiatrists’ Lead for Military and Veterans’ Health, a trustee of Walking with the Wounded, and an independent director at the Forces in Mind Trust; however, he was not directed by these organisations in any way in relation to his contribution to this paper. N.J. is a full-time member of the armed forces seconded to King's College London. N.T.F. reports grants from the US Department of Defense and the UK MoD, is a trustee (unpaid) of The Warrior Programme and an independent advisor to the Independent Group Advising on the Release of Data (IGARD). S.W. is a trustee (unpaid) of Combat Stress and Honorary Civilian Consultant Advisor in Psychiatry for the British Army (unpaid). S.W. is affiliated to the National Institute for Health Research Health Protection Research Unit (NIHR HPRU) in Emergency Preparedness and Response at King's College London in partnership with Public Health England, in collaboration with the University of East Anglia and Newcastle University. The views expressed are those of the author(s) and not necessarily those of the National Health Service, the NIHR, the Department of Health, Public Health England or the UK MoD.

Since 2001, more than 280 000 UK service personnel deployed to Iraq and Afghanistan, some on multiple occasions.^1^ Many of those who deployed are likely to have been exposed to potentially traumatic events. It is essential to ensure long-term follow-up of these individuals to understand the consequences of deployment, including once personnel have left military service.^2^^–^^5^ Research conducted among Vietnam veterans highlighted how concerns emerged about the mental health impact in the years following the conflict.^6^^–^^9^ The passage of time provides the opportunity to study what has happened to those who have left military service. More than 19 000 trained regular personnel leave the UK armed forces annually to resume civilian life.^10^ Research examining psychosocial outcomes for UK ex-serving personnel suggests that the majority transition with few difficulties;^11^^–^^13^ however, those who experience mental health difficulties during service appear to be at risk of developing social, occupational or health-related problems after leaving.^11^^,^^14^^,^^15^ In the UK, veterans are referred to as those who have left military service whereas in the USA, the term veteran is also applied to those who have served in a particular campaign (for example ‘Iraq veterans’). To prevent confusion, we will use the term ‘ex-serving personnel’ throughout to indicate former members of the UK armed forces.

In 2003, the King's Centre for Military Health Research established a cohort study to examine the potential impact of deployment to Iraq on the health and well-being of UK service personnel.^5^ So far findings have been reported on two waves of data collection (2004–2006 and 2007–2009) indicating that, at a population level, there was no overall excess of mental health consequences arising for those who deployed compared with other members of the armed forces who had not deployed to those conflicts.^4^^,^^5^ However, particular subgroups were found to be at an increased risk of mental ill health and alcohol misuse, namely reserves and those who experienced direct combat exposure. We now provide the main outcomes of the third wave of the cohort study. We describe the prevalence of mental disorders and alcohol misuse; investigate whether mental disorders differed between serving and ex-serving personnel and examine the association with deployment to Iraq and Afghanistan.

## Method

### Study design and participants

The cohort study started in June 2004, initially comparing the health of two randomly selected samples: individuals who had deployed to the initial conflict in Iraq (termed Op TELIC) were compared with individuals who were serving but who at that time had not deployed to Iraq (termed the Era group), despite being deployable. The study sought to recruit 17 698 individuals. Of the 17 499 actively followed up, 10 272 participated (a 59% response rate; these 10272 were included in the paper reporting on the outcomes of phase one^5^ and subsequently 37 responded late and were included in the follow-up).

Of the 9395 phase one participants who consented to further contact, 6429 (68%) completed phase two of the study that took place between November 2007 and September 2009. The original design had been based on the premise that this would be a replication of our previous studies on the impact of the 1991 Gulf War,^16^ but events made it necessary to modify this. The war in Iraq had continued, and UK troops had also been deployed to Afghanistan (termed Op HERRICK); two additional samples were needed in phase two. A random sample of personnel deployed to Afghanistan between April 2006 and April 2007 along with a new sample of trained regular and reserve personnel who had joined the service since April 2003 (the phase two replenishment sample). The final HERRICK sample comprised 1789 regular and reserve personnel of whom 896 (50%) responses were received. The final sample size of the replenishment sample was 6628 and of whom 2665 (40%) individuals responded. Overall, 9990 (56%) personnel responded at phase two. Full details of sampling and response rates have been reported previously.^4^ Subsequently, a short version of the phase two questionnaire was sent to all non-responders (*n* = 3109) and 300 (9.7%) responses were received.

Phase three re-contacted participants who consented to further contact in phases one or two, including those who completed a short version of the phase two survey (termed the follow-up sample). The follow-up sample comprised 12 280 individuals; 10 148 regular and 2132 reserve personnel. A replenishment sample of trained regular and reserve personnel who joined the military on or after 1 August 2009 and were still in service on the 31 March 2013 was also selected for sampling. This phase three replenishment sample comprised 8581 individuals, 6915 regular and 1666 reserve personnel (supplementary Fig. 1 available at https://doi.org/10.1192/bjp.2018.175).

### Procedures

Participation in phase three of the study involved completing a self-administered questionnaire that was available in both hard copy and electronic versions. Both versions of the questionnaire were piloted at an Army garrison to ensure that the questions were acceptable and understood. The questionnaire had sections covering (a) sociodemographics; (b) service information (which was completed by ex-serving personnel too), for example service branch, current or last rank, type of engagement, deployment status; (c) experiences during last deployment (for example duty, duration of deployment and combat exposure); (d) experiences of transition from deployment and home coming; (e) current mental and physical health; and (f) relationships and lifestyle (for example marital status, smoking and drinking behaviour).

Health questions enquired about symptoms of common mental disorders (CMD), using the 12-item General Health Questionnaire (GHQ-12);^17^ probable post-traumatic stress disorder (PTSD), using the 17-item National Centre for PTSD Checklist (PCL-C);^18^ and alcohol use, using the 10-item World Health Organization Alcohol Use Disorders Identification Test (AUDIT).^19^ Binary outcome variables were defined using the following cut-off scores for ‘caseness’: 4 or more for the GHQ-12 (scores range from 0 to 12),^20^ 50 or more for the PCL-C (scores range from 17 to 85)^18^ and 16 or more for the AUDIT (scores range from 0 to 40)^21^ (usually defined as hazardous use that is also harmful to health, which we have termed alcohol misuse).

### Data collection

Data collection took place between October 2014 and December 2016. Invitations were sent to individuals using addresses supplied by participants at earlier phases, or the address supplied by the UK Ministry of Defence if the person was in service or was newly sampled at phase three. Individuals were sent a postal invitation to complete the online survey along with a participant information leaflet. Non-responders to the postal invitation were sent a repeat invitation by email. Subsequently, all non-responders were sent a paper version of the questionnaire along with log-in details for the online version and a postage paid return envelope.

An intensive period of follow-up and tracing of non-responders began in September 2015 and continued until August 2016. For those who were still serving, key administrative personnel within the parent unit were approached to ask for assistance in distributing invitation packs and ascertaining new contact details for those who had moved unit. It was requested that, where possible, the potential participants be given allocated work time to complete the questionnaire. The voluntary nature of the study was emphasised; if individuals did not wish to take part they could return a blank questionnaire to the research team. A 5-day visit to a single large garrison was carried out in which a series of sessions were organised to enable participants to meet the members of the research team and complete a paper version of the questionnaire.

Those who had left service and reserves were contacted by telephone if they had provided home contact details before. Repeat invitation packs were mailed or emailed if an individual indicated that they were willing to take part. Where telephone numbers were incorrect, alternative numbers were sought from directory enquiries. Updated addresses were sought for individuals who were uncontactable by 25 August 2016 via the National Health Service Personal Demographics Service but only for those who had previously given consent to be re-contacted.

#### Analyses

All samples were combined; sample weights were generated to account for the different sampling groups used. Sample weights were calculated as the inverse probability of an individual being sampled from a specific subpopulation (TELIC, Era, HERRICK, phase two replenishment, phase three replenishment) and specific engagement type (regular or reserve). Response weights were generated to account for non-response and calculated as the inverse probability of responding once sampled, driven by factors shown empirically to predict response (gender, age, rank, service, engagement type, serving status, sample and the interaction between sample and engagement type). The sample weights and response weights were multiplied together to make a single weight. The weighted analyses provided valid results under the assumption that the data were missing at random and that the observed variables modelled to drive non-response were correctly identified.

The sociodemographic, military and health characteristics of our sample were described first using the full sample of the cohort, after which univariable and multivariable logistic regressions were carried out examining the association between deployment experiences and each mental disorder on subgroups of interest. The main analyses assessed the association between mental disorder (PTSD, CMD and alcohol misuse) and deployment experiences, stratified by engagement (regular or reserve personnel) and serving status (currently serving or ex-serving regular personnel). The primary exposure variables were deployed to Iraq and/or Afghanistan or not, role on last deployment (combat role or combat (service) support) and number of deployments. Self-reported deployment data were used. Weighted percentages and odds ratios (ORs) are presented in the tables along with unweighted cell counts. Multivariable analyses were adjusted for predefined sociodemographic (gender, age, marital and educational status) and military factors (rank, service, engagement type). All statistical analyses were performed using the statistical package Stata (version 14.2), with survey commands used to account for weighting.

### Ethical approval

Ethical approval for the study was granted by the UK Ministry of Defence Research Ethics Committee (reference: 448/MODREC/13) and the King's College London Psychiatry Nursing and Midwifery Research Ethics Subcommittee (Reference: PNM/12/13–169).

## Results

The overall adjusted response rate was 57.8% for the follow-up sample (*n* = 6346) and 24.0% for the replenishment sample (*n* = 1747) (overall response rate 44.3%) (supplementary Fig. 1). The features of non-response have not changed since the start of this cohort study: responders were significantly more likely to be older, women, regulars, hold officer rank, in the Royal Air Force rather than the Royal Navy and Royal Marines (henceforth together termed Naval Services) and the Army. An important finding is that poorer mental health at phase two did not predict response at phase three, suggesting that response was not biased by the main outcome except for those who reported symptoms of alcohol misuse at phase two who were less likely to respond (supplementary Table 1).

Table 1 describes the sociodemographic and military characteristics of those included in the study by deployment status. Of the 8093 participants included in the study, 61.9% had deployed to Iraq or Afghanistan. Those in the deployed group were in general more likely to be men, in the Army, regular personnel and currently serving. These factors are characteristic of the ground combat units and their supporting elements that make up the bulk of personnel during land deployments.

**Table 1:** Description of study participants (sociodemographic factors, military factors) by deployment status (total *n* = 8093)

	Not deployed (*n* = 2656, 38.1%)	Deployed^a^ (*n* = 5437, 61.9%)	*P*
Age group, years:^b^ *n* (%)			<0.001
<25	336 (6.3)	210 (2.7)	
25–29	457 (9.4)	530 (8.7)	
30–34	378 (13.8)	1130 (25.2)	
35–39	172 (7.6)	817 (16.2)	
40–49	643 (33.6)	1947 (36.7)	
>50	670 (29.3)	803 (10.5)	
Age, mean (s.d.)	39.97 (13.0)	40.20 (9.4)	
Gender (at baseline), *n* (%)			<0.001
Men	2210 (87.4)	4841 (91.7)	
Women	446 (12.6)	596 (8.3)	
Marital status, *n* (%)			0.66
In a relationship	2089 (85.5)	4485 (85.1)	
Not in a relationship	494 (14.5)	857 (14.9)	
Educational level, *n* (%)			0.15
Low (O levels/GCSEs or none)	779 (30.1)	1589 (32.1)	
High (A levels, degree or higher)	1862 (69.9)	3819 (67.9)	
Service (at baseline), *n* (%)			<0.001
Naval Services	686 (27.2)	589 (10.9)	
Army	1407 (51.2)	3762 (70.6)	
Royal Air Force	563 (21.5)	1086 (18.5)	
Rank, *n* (%)			<0.001
Officer	596 (18.9)	1557 (20.1)	
Non-commissioned officer	1331 (60.2)	3253 (66.8)	
Other rank	727 (20.8)	627 (13.1)	
Engagement type (at baseline), *n* (%)			<0.001
Regular	2060 (86.3)	4525 (92.0)	
Reserve	596 (13.7)	912 (8.0)	
Serving status, *n* (%)			<0.001
Serving	1384 (31.0)	3248 (50.0)	
Left service	1272 (69.0)	2189 (50.0)	

a. Deployed to Iraq or Afghanistan. Numbers are unweighted, frequencies are weighted.

b. The age distribution in the deployed group can be explained by the fact that the majority of those had deployed some time ago.

### Prevalence of mental disorders and alcohol misuse

Overall 21.9% (95% CI 20.75–23.01; *n* = 1739) of participants reported symptoms of CMD, 6.2% (95% CI 5.49–6.89; *n* = 417) reported probable PTSD and 10.0% (95% CI 9.20–10.90; *n* = 733) reported alcohol misuse. The prevalence of mental health outcomes varied by sociodemographic and military characteristics (supplementary Table 2).

### Type of engagement (regulars versus reserves)

As in phase one and phase two of the cohort study, there was a significant interaction between deployment status (not deployed to Iraq/Afghanistan or deployed to Iraq/Afghanistan) and engagement type (regular or reserve status) on CMD (*P* = 0.002) indicating a higher prevalence of CMD in deployed reserves compared with deployed regulars.^4^^,^^5^ No significant interaction was found for probable PTSD (*P* = 0.47) or alcohol misuse (*P* = 0.94). Still, because of the interaction we found for CMD, the impact of deployment was examined separately for regulars and reserves (Table 2). Compared with reserves who did not deploy to Iraq and/or Afghanistan, those reserves who had deployed continued to report significantly more symptoms of CMD, probable PTSD and alcohol misuse. Raised levels of PTSD were found among deployed regulars compared with those who had not deployed. Higher levels of alcohol misuse were reported in deployed regular personnel compared with those who had not deployed although the difference was not statistically significant (*P* = 0.063).

**Table 2:** Association between mental health outcomes and deployment status stratified by regular/reservist status

	Regulars				Reservists			
	Not deployed, *n* (%) (*n* = 2060, 36.6%)	Deployed,^a^ *n* (%) (*n* = 4525, 63.4%)	Odds ratio (95% CI)	Adjusted odds ratio^b^ (95% CI)	Not deployed, *n* (%) (*n* = 596, 51.2%)	Deployed,^a^ *n* (%) (*n* = 912, 48.8%)	Odds ratio (95% CI)	Adjusted odds ratio^b^ (95% CI)
Common mental disorders	443 (21.3)	961 (22.0)	1.04 (0.89–1.21)	1.01 (0.86–1.19)	106 (18.8)	229 (27.5)	**1.64 (1.20–2.25)**	**1.75 (1.26–2.44)**
Probable PTSD	104 (5.2)	245 (6.9)	**1.34 (1.00–1.78)**	**1.41 (1.04–1.90)**	15 (3.2)	53 (6.9)	**2.25 (1.14–4.46)**	**2.48 (1.20–5.16)**
Alcohol misuse	158 (8.3)	461 (11.4)	**1.42 (1.13–1.78)**	1.25 (0.99–1.57)	28 (5.5)	86 (9.9)	**1.88 (1.11–3.21)**	**1.74 (1.01–3.01)**

PTSD, post-traumatic stress disorder.

a. To Iraq and/or Afghanistan.

b. Adjusted for age (continuous), gender, marital status, educational status, service, rank.

As the majority of personnel who deployed constitute regulars and the number of reserves in our sample is relatively small, our analyses focused on the mental health of regulars in the next sections.

### Serving status (serving versus ex-serving)

In contrast to previous phases of the cohort study, our sample comprised a large number of ex-serving personnel. Table 3 shows the association between mental health outcomes according to serving status (currently serving and ex-serving). Findings showed that probable PTSD was lower among serving personnel than ex-serving personnel. After adjustment, probable PTSD and alcohol misuse were significantly associated with serving status: the odds were significantly higher in ex-serving personnel (adjusted OR = 1.73, 95% CI 1.25–2.40 and adjusted OR = 1.35, 95% CI 1.08–1.70, respectively). No difference was found in the prevalence of CMD by serving status.

**Table 3:** Association between mental health outcomes and serving status (regular personnel only)

	Serving, *n* (%) (*n* = 3887, 43.6%)	Ex-serving, *n* (%) (*n* = 2698, 56.4%)	Odds ratio (95% CI)	Adjusted odds ratio^a^ (95% CI)
Common mental disorders	849 (22.0)	555 (21.5)	0.97 (0.84–1.11)	1.06 (0.90–1.26)
Probable PTSD	170 (4.8)	179 (7.4)	**1.60 (1.25–2.06)**	**1.73 (1.25–2.40)**
Alcohol misuse	367 (10.2)	252 (10.3)	1.01 (0.83–1.23)	**1.35 (1.08–1.70)**

PTSD, post-traumatic stress disorder.

a. Adjusted for age (continuous), gender, marital status, educational status, service, rank and deployment to Iraq/Afghanistan.

We found a significant interaction between deployment status (not deployed to Iraq/Afghanistan or deployed to Iraq/Afghanistan) and serving status (serving or ex-serving) for probable PTSD (*P*<0.001) and alcohol misuse (*P*<0.01) indicating that the effect of deployment on PTSD and alcohol misuse differed in those still serving compared with those who had left service. There was no significant interaction for CMD (*P* = 0.09). So, because of the interaction for probable PTSD and alcohol misuse, the impact of deployment was examined separately by serving status (supplementary Table 3). Deployment was not statistically significantly associated with any of the mental health outcomes in serving regulars; the adjusted OR of alcohol misuse was slightly raised, but did not reach statistical significance (adjusted OR = 1.43, 95% CI 0.99–2.07; *P* = 0.06). In ex-serving regulars, probable PTSD remained significantly associated with deployment following adjustment (adjusted OR = 1.55, 95% CI 1.03–2.34). Alcohol misuse was no longer associated with deployment among ex-serving personnel in the adjusted analyses (adjusted OR = 1.19, 95% CI 0.86–1.64).

### Military role, number of deployments and time since deployment

To assess whether there was an association between experience of combat duties and mental health, further analysis was conducted among regular personnel who had been deployed to Iraq or Afghanistan, stratified by serving status. Overall around one-third held a combat role, whereas the majority were in a combat (service) support role such as medical, logistics, signals and aircrew. In contrast to previous results from the cohort study, no association was found between self-reported role and the mental health outcomes or alcohol misuse among serving regulars (Table 4). PTSD was higher in those with combat exposure, but not sufficiently robust for statistical inference.

**Table 4:** Association between mental health outcomes and self-reported role during last deployment among regular personnel, stratified by serving status (serving or ex-serving)

	Serving regulars				Ex-serving regulars			
	Combat (service) support role, *n* (%) (*n* = 1990, 70.9%)	Combat, *n* (%) (*n* = 812, 29.1%)	Odds ratio (95% CI)	Adjusted odds ratio^a^ (95% CI)	Combat (service) support role, *n* (%) (*n* = 1224, 67.5%)	Combat, *n* (%) (*n* = 495, 32.5%)	Odds ratio (95% CI)	Adjusted odds ratio^a^ (95% CI)
Common mental disorders	424 (21.9)	161 (19.7)	0.88 (0.69–1.11)	0.97 (0.74–1.26)	228 (18.8)	148 (30.7)	**1.91 (1.44–2.53)**	**1.70 (1.28–2.41)**
Probable PTSD	60 (3.7)	48 (6.1)	1.70 (1.08–2.67)	1.58 (0.98–2.55)	60 (5.7)	77 (17.1)	**3.39 (2.25–5.11)**	**2.53 (1.60–3.99)**
Alcohol misuse	190 (10.4)	94 (12.0)	1.17 (0.86–1.58)	0.99 (0.71–1.38)	107 (10.6)	70 (14.9)	**1.48 (1.02–2.16)**	1.08 (0.72–1.65)

PTSD, post-traumatic stress disorder.

a. Adjusted for age (continuous), gender, marital status, educational status, service, rank.

For ex-serving regulars, unadjusted analyses suggested that those who held a combat role were more likely to report symptoms of CMD, probable PTSD and alcohol misuse compared with those who deployed in a combat (service) support role. After adjustment, these associations remained significant for CMD and PTSD (adjusted OR = 1.70, 95% CI 1.28–2.41 and adjusted OR 2.53, 95% CI 1.60–3.99, respectively) (Table 4).

No association was found between the number of deployments and mental health problems, irrespective of serving status among Army and Royal Marines, who provide the majority of personnel during land combat operations (supplementary Tables 4 and 5).

### Comparison across cohort study phases

The adjusted prevalence estimates of mental health outcomes and alcohol misuse across the three phases of the cohort study among serving regular and ex-serving regular personnel can be found in supplementary Tables 6 and 7.

A reduction in alcohol misuse is apparent in both regular serving and ex-serving personnel, especially when comparing rates in deployed personnel from phase three with phase two. Over time, rates of probable PTSD seem to be relatively stable in serving personnel. However, increased rates are found in ex-serving regular personnel, irrespective of whether they have been deployed or not. Levels of CMD symptoms have decreased over time in ex-serving regulars whereas CMD are now more common among serving personnel in phase three, compared with the other two phases, with the highest levels found among those not deployed.

## Discussion

The third phase of this UK military cohort study showed that of the three main mental health problems measured, CMD and alcohol misuse continue to be the most common mental health problems reported by UK service personnel. Ex-serving regulars were reporting higher levels of PTSD and alcohol misuse than serving regulars. Further, past deployment to Iraq or Afghanistan was associated with higher rates of probable PTSD among ex-serving regulars. The highest levels of PTSD and CMD were found in ex-serving regulars who had deployed in a combat role. Rates of alcohol misuse were comparable, irrespective of serving status. Neither history of deployment nor last deployment in a combat role was associated with adverse mental health outcomes or alcohol misuse in serving regulars.

### Strengths and limitations

First, non-response rates and characteristics have not changed since the start of the study, and are intrinsic to all studies of this type of population.^22^ The response rate of the replenishment group was disappointing in contrast to the follow-up group. We made extensive efforts using multiple methods of tracing and recruiting participants, but this group was more reluctant to participate than the rest of the cohort. We applied adequate weighing procedures to mitigate this potential source of bias as explained in the methods section. We are not alone in reporting difficulties to recruit military personnel in large studies. The response rate of the US Millennium Cohort was 77 058 (30%) out of a total sample of 256 400. The RAND report *Invisible Wounds of War* also received a very limited response, however, the actual rate was difficult to estimate because the sampling frame included many telephone numbers that were not working.^23^^,^^24^ Poor mental health at phase two did not predict response at phase three, with the exception of alcohol misuse and even for alcohol misuse this association was mild. Second, individuals with probable mental disorders were identified using self-report measures, still these have been validated and used extensively in general population studies. Further, these measures have been used in the previous phases of the cohort study and hence enable comparisons over time. Third, the analyses presented here are cross-sectional and therefore causal interpretations cannot be drawn. Nevertheless, the results of this study are based on large, randomly selected samples of deployed and not deployed UK military serving personnel representing the UK armed forces participation in Iraq and Afghanistan conflicts throughout the whole period, i.e. from 2003 until 2015.

### Differences in mental health outcomes and alcohol misuse among serving and ex-serving regular personnel

The prevalence of probable PTSD in previously deployed ex-serving regular personnel was found to be more than twice the rate found among previously deployed serving regulars (9% versus 4%) (supplementary Table 3). The differential impact of deployment on mental health outcomes in current and former personnel becomes even more pronounced when examined in relation to deploying in a combat role. In contrast to the previous phases of the cohort study, no association was found between combat role and poorer mental health in serving regular personnel; however among ex-serving regulars symptoms of probable PTSD (17% versus 6%) and CMD (31% versus 19%) were significantly elevated.^4^^,^^5^ A possible explanation is that personnel who are mentally unwell either elect to leave service or are more likely to be discharged for medical reasons and also have poorer outcomes after leaving service.^11^^,^^25^ Self-reported role during the last deployment was used as a proxy for combat exposure. We admit that those who reported being in a combat (service) support role may still have been exposed to combat, for example those working in medical services or counter-improvised explosive device teams.^26^ A systematic review suggested that combat-related PTSD was most strongly associated with having experienced combat, discharging a weapon and having seen someone wounded or killed.^27^^–^^29^ Although, self-reported combat role may not have been totally reflective of the range of specific combat experiences personnel may have been exposed to, it is a good summary measure of combat exposure during deployment. Further, we have used the same measure when describing the results of the previous phases of this cohort study thereby allowing the comparison of findings.^4^^,^^5^ Although in a military context combat exposure is the main factor under consideration, other factors could be associated with the development of PTSD that may not be related to deployment such as childhood adversity, traffic road accidents, sexual abuse and other additional life stresses.^30^

A recent international systematic review indicated that a minority of personnel who leave the Services experience hardship in a range of life domains such as homelessness, financial difficulties, psychological difficulties, substance misuse and lack of social support.^31^ It is possible that, for some, the transition from military service to civilian life triggers additional stresses that can have a negative impact on mental health. In addition, personnel may be more reluctant to disclose their symptoms during service, as they may fear being stigmatised or the possibility of negative occupational consequences should they report mental health symptoms.^32^^,^^33^

As shown in the previous phases of the cohort study, for Army and Royal Marine regular personnel, no association was found between the number of deployments undertaken to Iraq or Afghanistan and any mental health outcomes, irrespective of remaining in Service or leaving.^4^^,^^5^ This finding could be attributed in part to the healthy warrior concept; a selection effect whereby those who are unwell are less likely to deploy again or are more likely to leave Service.^34^ Alternatively, the lack of association between number of deployments and poorer mental health may represent a positive effect arising from adherence to the UK Armed Forces Harmony Guidelines that recommend both limiting the cumulative frequency of deployment and adhering to the suggested length of time between deployments.^35^ However, we did not account for length of deployment in our analyses or the time between deployments. The reason for this decision was that we have published two papers that examined this specifically.^35^^,^^36^ The cohort study primarily examined the health and well-being legacy of deployment to Iraq and Afghanistan; it is possible that various other concurrent operations may have contributed to the mental health impact of deployment.

### Differences in mental health outcomes and alcohol misuse among deployed and non-deployed reserves

Mental health symptomatology of deployed reserves is comparable with that of deployed regulars. However, when we compare deployed reserves with reserves who did not deploy, they reported worse health outcomes. These findings have been replicated over the duration of the cohort study. There are various explanations why we see a deployment effect for reserves, for example more problematic homecoming experiences and more limited support or understanding from reserve families and close friends about aspects of military life and deployment than that found among regulars.^37^ Furthermore, reserves tend to deploy as singletons or small specialist units and as such do not have access to the supportive structure of formed units pre- and post-deployment.

### Prevalence rates of CMD, PTSD and alcohol misuse across cohort phases

The overall prevalence of CMD remained similar across the three phases of the cohort study whereas the prevalence of alcohol misuse declined at each phase, irrespective of serving status.^4^^,^^5^ In contrast to phase two results of the cohort study, alcohol misuse was no longer associated with deployment at phase three. There are three major factors that could account for the decline: first, there has been a comparable decline in alcohol consumption in the UK general population;^38^ second, alcohol misuse becomes less frequent with age and, although a replenishment sample was recruited to minimise this effect, the average age of cohort participants has increased over time; third, alcohol misuse at phase two was associated with non-response during phase three; however, this association was mild.

Probable PTSD remained the least common outcome compared with CMD, but the overall prevalence of probable PTSD increased from 4 to 6% between phase one/two and phase three. In previous phases of the cohort study, rates of PTSD in the military population were overall similar to the general population, but now appear to be elevated by comparison.^39^ This seems to be mainly driven by the higher rates found in ex-serving personnel, particularly those who deployed in a combat role. However, the levels found in the UK military have yet to reach the levels seen in the US military^40^^–^^42^ and do not as of yet seem to justify ‘bow wave’, ‘tsunami’ or ‘time bomb’ metaphors commonly reported in the popular press, although of course this can always change.

Levels of PTSD have repeatedly been shown to be lower in the UK military compared with the USA and various explanations have been put forward concerning deployment characteristics, such as possible higher levels of combat exposure among US troops, differential demographics of those deployed (US personnel more likely to be younger, of lower rank and reservists), and length of deployment as well as cultural differences such as access to healthcare and attitudes towards trauma reporting.^26^ Still, the increase of PTSD rates in ex-serving personnel stresses the importance of continued surveillance.

The latest Adult Psychiatric Morbidity Survey (APMS) completed in 2014, a household survey investigating the mental health of the UK adult population, suggests that general population levels of PTSD are around 4.4%. Rates remained stable for men, whereas a sharp decrease was found in women after the age of 24.^43^ This is contrary to the findings among UK military personnel, as we found higher rates of PTSD in ex-serving personnel, who tend to be older than those currently serving; this could represent a deployment effect that would not be found among the general population. Further, men reported higher levels of alcohol misuse than women. This finding is in line with the APMS study that indicated that men were drinking at more hazardous levels compared with women.^43^ Women reported greater symptoms of CMD (24.3% versus 21.6%) than men but this difference did not reach statistical significance (supplementary Table 2). This is in contrast with previous studies investigating gender differences related to mental health outcomes among military personnel and in the general population, whereby CMD are found to be more prevalent in women than men.^44^^,^^45^

### Implications

Alcohol misuse and CMD continue to be the most common mental health conditions among UK serving and ex-serving personnel. In general, the prevalence of PTSD has increased from 2004/6. This appears to be related to higher rates of PTSD among ex-serving personnel in the latest cohort phase. In addition, ex-serving personnel are experiencing higher rates of CMD compared with serving personnel, suggesting that the risk of mental ill health is carried by those who have left the service. Therefore, our results support the current focus on providing and improving veteran mental health services. The UK Ministry of Defence seeks to ensure that, after personnel join, train and work well, they also leave well, transition smoothly to civilian life and live well after service.^46^ The study data suggests that transition and civilian life may be particularly problematic for an important minority of UK Service personnel. As seen previously with regards to mental health legacy issues, such as those from the Vietnam War, these have taken some time to reveal themselves. These results reiterate the importance of taking a lifelong approach to the health and well-being of the armed forces to ensure that both serving and ex-serving personnel receive optimal care.

## Supplementary material

For supplementary material accompanying this paper visit https://doi.org/10.1192/bjp.2018.175. click here to view supplementary material
