# Mental health outcomes at the end of the British involvement in the Iraq and Afghanistan conflicts: a cohort study – CORRIGENDUM

**DOI:** 10.1192/bjp.2018.235

**Published:** 2019-03

**Authors:** Sharon A. M. Stevelink, Margaret Jones, Lisa Hull, David Pernet, Shirlee MacCrimmon, Laura Goodwin, Deirdre MacManus, Dominic Murphy, Norman Jones, Neil Greenberg, Roberto J. Rona, Nicola T. Fear, Simon Wessely

The original version of this paper contained an omission in the conflict of interest statement. The correct version of the conflict of interest statement is below:

All authors are based at King's College London which, for the purpose of this study and other military-related studies, receives funding from the UK Ministry of Defence (MoD). S.A.M.S., M.J., L.H., D.P., S.M. and R.J.R. salaries were totally or partially paid by the UK MoD. The UK MoD provides support to the Academic Department of Military Mental Health, and the salaries of N.J., N.G. and N.T.F. are covered totally or partly by this contribution. D.Mu. is employed by Combat Stress, a national UK charity that provides clinical mental health services to veterans. D.MacM. is the lead consultant for an NHS Veteran Mental Health Service. N.G. is the Royal College of Psychiatrists’ Lead for Military and Veterans’ Health, a trustee of Walking with the Wounded, and an independent director at the Forces in Mind Trust; however, he was not directed by these organisations in any way in relation to his contribution to this paper. N.J. is a full-time member of the armed forces seconded to King's College London. N.T.F. reports grants from the US Department of Defense and the UK MoD, is a trustee (unpaid) of The Warrior Programme and an independent advisor to the Independent Group Advising on the Release of Data (IGARD). S.W. is a trustee (unpaid) of Combat Stress and Honorary Civilian Consultant Advisor in Psychiatry for the British Army (unpaid). S.W. is affiliated to the National Institute for Health Research Health Protection Research Unit (NIHR HPRU) in Emergency Preparedness and Response at King's College London in partnership with Public Health England, in collaboration with the University of East Anglia and Newcastle University.

This paper represents independent research part funded by the National Institute for Health Research (NIHR) Biomedical Research Centre at South London and Maudsley NHS Foundation Trust and King's College London.

The views expressed are those of the author(s) and not necessarily those of the National Health Service, the NIHR, the Department of Health and Social Care, Public Health England or the UK MoD.

## References

[ref1] Stevelink SAM, Jones M, Hull L, Pernet D, MacCrimmon S, Goodwin L, Mental health outcomes at the end of the British involvement in the Iraq and Afghanistan conflicts: a cohort study. The British Journal of Psychiatry 2018; 1–8. 10.1192/bjp.2018.175 PMC6429255 30295216

