# Supplementary material for: Mental health outcomes at the end of the British involvement in the Iraq and Afghanistan conflicts: a cohort study
Source: Br J Psychiatry. 2018 Dec;213(6):690–7. doi: 10.1192/bjp.2018.175 (PMC6429255; doi:10.1192/bjp.2018.175)
Supplement: Supplementary file 1 [file S0007125018001757sup001.zip › S0007125018001757sup001/Summary sampling and response figure v2.docx]

Supplementary Figure 1: Summary of sampling and response

**Phase 2 follow up sample**

**Regulars n=8686**

**Reserves n=1586**

**P1 late responders**

**Removed***

**Attempted to contact at P2**

**P2 late responders**

**Completed P2**

**Removed***

**Responded at P1 not P2/ completed a P2 short questionnaire**

**+32**

**Contacted for P3**

**Completed P3**

**N=7884**

**N=1511**

**N=5334**

**N=1095**

**+31**

**-176**

**+2375**

**N=7564**

**N=3948**

**N=1497**

**N=902**

**+5**

**-80**

**+5**

**-10**

**+407**

**Phase 2 HERRICK sample**

**Phase 2 Replenishment sample**

**Regulars n=1491**

**Regulars n=5583**

**Reserves n=334**

**Reserves n=1855**

**-36**

**-455**

**-355**

**N=1455**

**N=746**

**N=334**

**N=150**

**N=5128**

**N=2198**

**N=1500**

**N=467**

**+3**

**+1**

**+15**

**+3**

**-138**

**-10**

**-492**

**-51**

**+65**

**+16**

**+187**

**+59**

**N=676**

**N=157**

**N=1908**

**N=478**

**N=362**

**N=94**

**N=780**

**N=260**

**P3 Replenishment sample**

**N=6915**

**N=1666**

**N=1495**

**N=252**

**Regulars n=7108**

**Reserves n=1680**

**-14**

**-193**

**- 834**

**Phase 1 responders**

*Participants were removed because they said no to future contacts, had died, were found to be ineligible, or had insufficient address information.

**P1 = phase 1, P2 = phase 2, P3 = phase 3**
