# Supplementary material for: Mental health outcomes at the end of the British involvement in the Iraq and Afghanistan conflicts: a cohort study
Source: Br J Psychiatry. 2018 Dec;213(6):690–7. doi: 10.1192/bjp.2018.175 (PMC6429255; doi:10.1192/bjp.2018.175)
Supplement: Supplementary file 1 [file S0007125018001757sup001.zip › S0007125018001757sup001/Supplementary Tables v2.docx]

Supplementary Table 1: Characteristics of responders and non-responders contacted at phase 3 (including phase 3 late responders)

*Number attempted to contact n=20,861*

|  | Number (%) not responding | Number (%) responding | Adjusted* OR of responding |
| --- | --- | --- | --- |
| **Age group (years)** |  |  |  |
| <25 | 3671 (81.9) | 812 (18.1) | 1.00 |
| 25-29 | 2864 (74.5) | 982 (25.5) | 1.11 (0.99-1.24) |
| 30-34 | 2206 (63.9) | 1244 (36.1) | 1.25 (1.09-1.43) |
| 35-39 | 1320 (52.5) | 1196 (47.5) | 1.61 (1.38-1.88) |
| 40-49 | 1933 (45.2) | 2346 (54.8) | 2.35 (2.01-2.74) |
| >50 | 774 (33.8) | 1513 (66.2) | 3.93 (3.29-4.70) |
| **Sex (at baseline)** |  |  |  |
| Male | 11562 (62.1) | 7051 (37.9) | 1.00 |
| Female | 1206 (53.7) | 1042 (46.4) | 1.51 (1.37-1.67) |
| **Engagement type (at baseline)** |  |  |  |
| Regular | 10478 (61.3) | 6585 (38.6) | 1.00 |
| Reserve | 2290 (60.3) | 1508 (39.7) | 0.89 (0.82-0.97) |
| **Rank** |  |  |  |
| Other rank | 11694 (66.3) | 5956 (33.8) | 1.00 |
| Officer | 1074 (33.5) | 2137 (66.6) | 2.03 (1.86-2.22) |
| **Service**  **(at baseline)** |  |  |  |
| Naval  services | 2078 (62.0) | 1274 (38.0) | 0.96 (0.88-1.04) |
| Army | 8747 (62.9) | 5170 (37.2) | 1.00 |
| RAF | 1943 (54.1) | 1649 (45.9) | 1.14 (1.05-1.24) |
| **Serving status** |  |  |  |
| Serving | 6206 (58.7) | 4361 (41.3) | 1.00 |
| Left Service | 6562 (63.8) | 3732 (36.3) | 0.40 (0.38-0.43) |
| **Sample** |  |  |  |
| P2 follow up | 4211 (46.5) | 4850 (53.5) | 1.00 |
| P2 Herrick & P2 replenishment | 1723 (53.5) | 1496 (46.5) | 0.97 (0.88-1.08) |
| P3  replenishment | 6834 (79.6) | 1747 (20.4) | 0.33 (0.29-0.38) |
| **Phase 2 health for phase 3 follow up sample only** n=9169 | | | |
| **Symptoms of common mental disorders** | 749 (20.6) | 1021 (18.8) | 1.02 (0.91-1.14) |
| **Probable PTSD** | 179 (4.9) | 171 (3.2) | 0.84 (0.67-1.05) |
| **Hazardous drinking** | 2192 (60.5) | 2890 (53.2) | 0.95 (0.87-1.04) |
| **Alcohol misuse** | 609 (16.8) | 574 (10.6) | 0.83 (0.73-0.95) |
| **Multiple symptoms** | 373 (10.2) | 506 (9.3) | 1.02 (0.88-1.19) |

RAF=Royal Air Force. OR=Odds Ratio. P-value based on Chi squared test. Numbers are unweighted, frequencies are weighted. *adjusted for all other variables in the table but not for Phase 2 health variables. Hazardous drinking defined as scoring 8 or more on the Alcohol Use Disorders Identification Test (AUDIT). Alcohol misuse defined as scoring 16 or more for the AUDIT (usually defined as hazardous use that is also harmful for health). Multiple symptoms case defined as reporting 18 or more symptoms (out of a total of 53).

Supplementary Table 2: Prevalence of mental health outcomes by socio-demographic, military factors and deployment history

|  | Common mental disorders  n=1739 (21.9%)  n (%) | p value | Probable PTSD  n=417 (6.2%)  n (%) | p value | Alcohol misuse  n=733 (10.0%)  n (%) | p value |
| --- | --- | --- | --- | --- | --- | --- |
| **Age group (years)** |  |  |  |  |  |  |
| <25 | 131 (26.2) |  | 32 (7.0) |  | 77 (15.5) |  |
| 25-29 | 232 (25.2) |  | 53 (6.4) |  | 89 (11.7) |  |
| 30-34 | 363 (24.8) |  | 88 (7.7) |  | 158 (12.1) |  |
| 35-39 | 229 (22.5) |  | 62 (7.6) |  | 95 (11.0) |  |
| 40-49 | 547 (21.8) |  | 134 (6.1) |  | 220 (9.4) |  |
| >50 | 237 (15.4) | <0.001 | 48 (2.9) | <0.001 | 94 (6.1) | <0.001 |
| **Sex** |  |  |  |  |  |  |
| Male | 1487 (21.6) |  | 365 (6.2) |  | 677 (10.6) |  |
| Female | 252 (24.3) | 0.12 | 52 (5.9) | 0.83 | 56 (4.8) | <0.001 |
| **Marital status** |  |  |  |  |  |  |
| In a relationship | 1338 (20.2) |  | 292 (5.3) |  | 575 (9.4) |  |
| Not in a relationship | 382 (31.0) | <0.001 | 120 (11.0) | <0.001 | 156 (13.4) | <0.001 |
| **Service** |  |  |  |  |  |  |
| Naval Services | 252 (19.6) |  | 59 (5.3) |  | 108 (9.2) |  |
| Army | 1117 (22.5) |  | 306 (7.2) |  | 506 (10.9) |  |
| RAF | 370 (21.9) | 0.20 | 52 (3.5) | <0.001 | 119 (7.8) | 0.02 |
| **Rank** |  |  |  |  |  |  |
| Officer | 391 (18.7) |  | 59 (3.3) |  | 162 (8.1) |  |
| NCO | 951 (20.6) |  | 229 (5.5) |  | 439 (10.3) |  |
| Other rank | 397 (30.8) | <0.001 | 129 (12.2) | <0.001 | 132 (11.4) | 0.05 |
| **Engagement type** |  |  |  |  |  |  |
| Regular | 1404 (21.7) |  | 349 (6.3) |  | 619 (10.3) |  |
| Reserve | 335 (23.1) | 0.34 | 68 (5.1) | 0.17 | 114 (7.7) | 0.02 |
| **Deployed to Iraq and/or Afghanistan** |  |  |  |  |  |  |
| No | 549 (21.0) |  | 119 (5.0) |  | 186 (7.9) |  |
| Yes | 1190 (22.4) | 0.24 | 298 (6.9) | 0.01 | 547 (11.3) | <0.001 |
| **Serving status** |  |  |  |  |  |  |
| Serving | 1002 (22.0) |  | 191 (4.6) |  | 415 (9.9) |  |
| Left Service | 737 (21.8) | 0.87 | 226 (7.3) | <0.001 | 318 (10.1) | 0.74 |
| **Sample** |  |  |  |  |  |  |
| Follow-up | 1313 (21.3) |  | 327 (6.1) |  | 565 (9.9) |  |
| Replenishment | 426 (26.2) | <0.001 | 90 (6.3) | 0.81 | 168 (11.3) | 0.14 |

RAF=Royal Air Force. NCO=Non-Commissioned Officer. Numbers are unweighted, frequencies are weighted. P-value based on Chi square test.

Supplementary Table 3: Association between mental health outcomes and deployment status among regular personnel, stratified by serving status (serving or ex-serving)

|  | **Serving regulars** | | | | **Ex-serving regulars** | | | |
| --- | --- | --- | --- | --- | --- | --- | --- | --- |
|  | Not deployed  n=1081 (26.1%)  n (%) | Deployed**  n=2806 (73.9%)  n (%) | Odds ratio (95%CI) | Adjusted odds ratio* (95%CI) | Not deployed  n=979 (44.7%)  n (%) | Deployed**  n=1719  (55.3%)  n (%) | Odds ratio (95%CI) | Adjusted odds ratio* (95%CI) |
| Common mental disorders | 264 (24.4) | 585 (21.2) | 0.84 (0.69-1.02) | 0.94 (0.75-1.18) | 179 (19.9) | 376 (22.7) | 1.18 (0.95-1.47) | 0.98 (0.77-1.25) |
| Probable PTSD | 62 (5.8) | 108 (4.4) | 0.74 (0.50-1.09) | 0.89 (0.59-1.33) | 42 (5.0) | 137 (9.4) | **2.00 (1.35-2.95)** | **1.55 (1.03-2.34)** |
| Alcohol misuse | 83 (8.4) | 284 (10.9) | 1.32 (0.97-1.80) | 1.43 (0.99-2.07) | 75 (8.3) | 177 (12.0) | **1.51 (1.11-2.06)** | 1.19 (0.86-1.64) |

* adjusted for age (continuous), sex, marital status, educational status, Service, rank.

** To Iraq and/or Afghanistan

Supplementary Table 4: Association between mental health outcomes and number of deployments to Iraq or Afghanistan, in currently serving regular army or Royal Marine personnel only

|  | 1 deployment  n=803 (35.9%)  n (%) | 2 deployments  n=530 (28.4%)  n (%) | Odds ratio (95% CI) | Adjusted odds ratio* (95% CI) | 3 or more deployments n=674 (35.7%)  n (%) | Odds ratio (95% CI) | Adjusted odds ratio* (95% CI) |
| --- | --- | --- | --- | --- | --- | --- | --- |
| Common mental disorders | 158 (21.5) | 95 (17.2) | 0.76 (0.55-1.05) | 0.75 (0.53-1.06) | 138 (21.2) | 0.98 (0.73-1.32) | 0.99 (0.71-1.38) |
| Probable PTSD | 33 (5.6) | 24 (5.9) | 1.07 (0.58-1.98) | 1.06 (0.55-2.04) | 30 (4.2) | 0.75 (0.42-1.34) | 0.78 (0.40-1.51) |
| Alcohol misuse | 80 (10.6) | 55 (10.6) | 1.00 (0.66-1.52) | 1.31 (0.82-2.08) | 67 (11.4) | 1.09 (0.73-1.62) | 1.35 (0.85-2.16) |

adjusted for age (continuous), sex, marital status, educational status, rank

Supplementary Table 5: Association between mental health outcomes and number of deployments to Iraq or Afghanistan, in ex-serving regular army or Royal Marine personnel only

|  | 1 deployment  n=627 (51.1%)  n (%) | 2 deployments  n=369 (32.6%)  n (%) | Odds ratio (95% CI) | Adjusted odds ratio* (95% CI) | 3 or more deployments n=198 (16.3%)  n (%) | Odds ratio (95% CI) | Adjusted odds ratio* (95% CI) |
| --- | --- | --- | --- | --- | --- | --- | --- |
| Common mental disorders | 133 (22.7) | 97 (27.2) | 1.27 (0.90-1.81) | 1.34 (0.93-1.92) | 46 (21.2) | 0.92 (0.58-1.45) | 0.88 (0.55-1.40) |
| Probable PTSD | 58 (11.3) | 35 (10.7) | 0.94 (0.57-1.57) | 1.06 (0.62-1.79) | 21 (10.2) | 0.89 (0.49-1.62) | 0.85 (0.45-1.61) |
| Alcohol misuse | 65 (12.7) | 45 (13.4) | 1.07 (0.67-1.71) | 0.93 (0.58-1.51) | 26 (14.9) | 1.21 (0.68-2.15) | 1.03 (0.57-1.86) |

adjusted for age (continuous), sex, marital status, educational status, rank

Supplementary Table 6: Association between mental health outcomes and deployment status at the three phases of the cohort study in serving regulars only

|  | Phase 1 (2004-2006) | | | Phase 2 (2007-2009) | | | Phase 3 (2014-2016) | | |
| --- | --- | --- | --- | --- | --- | --- | --- | --- | --- |
|  | Era  n=4172 (53.7%) n (%) | Telic 1  n=3594 (46.3%) n (%) | Adjusted odds ratio# (95% CI) | Not deployed  n=2452 (41.3%)  n (%) | Deployed**  n=4061 (58.7%)  n (%) | Adjusted odds ratio## (95% CI) | Not deployed  n=1081 (26.1%)  n (%) | Deployed**  n=2806 (73.9%)  n (%) | Adjusted odds ratio## (95% CI) |
| Common mental disorders | 796 (19.3) | 643 (18.3) | 0.97 (0.86-1.10) | 477 (19.7) | 715 (18.5) | 0.90 (0.76-1.06) | 264 (24.4) | 585 (21.2) | 0.94 (0.75-1.18) |
| Probable PTSD | 131 (3.2) | 120 (3.4) | 1.08 (0.83-1.40) | 73 (2.9) | 122 (3.1) | 0.85 (0.58-1.24) | 62 (5.8) | 108 (4.4) | 0.89 (0.59-1.33) |
| Alcohol misuse | 576 (13.9) | 631 (17.8) | **1.17 (1.03-1.34)** | 280 (10.9) | 666 (15.6) | **1.26 (1.03-1.53)** | 83 (8.4) | 284 (10.9) | 1.43 (0.99-2.07) |

Sample and response weights have been applied for each phase where appropriate. Numbers are unweighted, frequencies are weighted.

#adjusted for age, sex, rank, educational and marital status, service brank and fitness to deploy

** To Iraq and/or Afghanistan

## adjusted for age, sex, rank, educational and marital status and service branch

Supplementary Table 7: Association between mental health outcomes and deployment status at the three phases of the cohort study in ex-serving regulars only

|  | Phase 1 |  |  | Phase 2 |  |  | Phase 3 |  |  |
| --- | --- | --- | --- | --- | --- | --- | --- | --- | --- |
|  | Era  n=550 (62.9%) n (%) | Telic 1  n=324 (37.1%) n (%) | Adjusted odds ratio# (95% CI) | Not deployed  n=1318 (79.4)  n (%) | Deployed**  n=437 (20.6)  n (%) | Adjusted odds ratio## (95% CI) | Not deployed  n=979 (44.7)  n (%) | Deployed**  n=1719 (55.3)  n (%) | Adjusted odds ratio## (95% CI) |
| Common mental disorders | 142 (26.1) | 101 (32.1) | 1.24 (0.88-1.75) | 269 (20.9) | 110 (26.9) | 1.21 (0.87-1.69) | 179 (19.9) | 376 (22.7) | 0.98 (0.77-1.25) |
| Probable PTSD | 39 (7.2) | 34 (10.8) | 1.52 (0.89-2.60) | 78 (6.0) | 48 (11.9) | **1.73 (1.06-2.84)** | 42 (5.0) | 137 (9.4) | **1.55 (1.03-2.34)** |
| Alcohol misuse | 80 (14.7) | 64 (20.3) | 0.97 (0.63-1.49) | 145 (11.3) | 89 (23.0) | **1.65 (1.12-2.42)** | 75 (8.3) | 177 (12.0) | 1.19 (0.86-1.64) |

Sample and response weights have been applied for each phase where appropriate. Numbers are unweighted, frequencies are weighted.

#adjusted for age, sex, rank, educational and marital status, service branch and fitness to deploy

** To Iraq and/or Afghanistan

## adjusted for age, sex, rank, educational and marital status and service branch
